# The Mite *Steatonyssus periblepharus* Is a Novel Potential Vector of the Bat Parasite *Trypanosoma dionisii*

**DOI:** 10.3390/microorganisms11122906

**Published:** 2023-12-01

**Authors:** Marina N. Malysheva, Anna I. Ganyukova, Alexander O. Frolov, Dmitriy V. Chistyakov, Alexei Yu. Kostygov

**Affiliations:** 1 Zoological Institute of the Russian Academy of Sciences, 199034 St. Petersburg, Russia; malmarnik@yandex.ru (M.N.M.); anna.ganyukova@gmail.com (A.I.G.); frolal@yandex.ru (A.O.F.); 2 Department of Vertebrate Zoology, Faculty of Biology, St. Petersburg State University, 199034 St. Petersburg, Russia; batsnwr@mail.ru

**Keywords:** Chiroptera, bats, trypanosome, 18S rRNA gene, developmental stages, life cycle

## Abstract

*Trypanosoma dionisii*, for which only bat bugs (Cimicidae) had previously been demonstrated as vectors, was, for the first time, detected in the gamasine mite *Steatonyssus periblepharus* in Russia. The molecular phylogenetic analysis indicated that trypanosomes found in these mites belong to the “clade A” of *T. dionisii*, which, based on genetic distances, can be considered as a species separate from the sister clade B, and according to available data also has a distinct geographic distribution. The presence of developmental forms of *T. dionisii* resembling those previously described during the development of this trypanosome in cimicids suggests that *S. periblepharus* is a novel vector of the studied trypanosome.

## 1. Introduction

Chiroptera (bats) are the second largest order of mammals after rodents [1]. They are widely distributed throughout the world and often live close to humans. Therefore, these animals represent important reservoir hosts for various pathogens, including trypanosomes [2,3]. To date, about 40 *Trypanosoma* spp. have been detected in bats [3,4]. Nevertheless, the majority of these species are known only by morphological descriptions. According to molecular records, the trypanosomes from Chiroptera predominantly belong to the so-called “*Trypanosoma cruzi* clade”, which includes the subgenera *Schizotrypanum* and *Aneza*, the *T. wauwau* clade, as well as *T. livingstonei* [3].

The most practically important trypanosome species that inhabits bats is *T. cruzi*, the agent of the dangerous Chagas disease in humans [5,6]. It has been suggested that the trypanosomes ancestral to *T. cruzi* were originally parasites of bats, from which they successfully switched to other mammalian hosts in both the New World and the Old World [3,6,7,8]. A number of peculiarities of the chiropteran biology, in particular their propensity to form colonies and their ability to migrate long distances, have apparently considerably contributed to the spread of such trypanosomes.

The most common trypanosome detected in bats is the globally distributed species *T. dionisii* [5,8,9], which is a close relative of *T. cruzi* and, as such, belongs to the same subgenus *Schizotrypanum*. For a long time, it was thought to occur only in bats, but recent studies have shown that this species can also infect other mammals, such as opossums and some carnivores [10]. In addition, there was a single documented case of human infection, when *T. dionisii* was revealed in the heart muscle tissue of a child who died of Chagas disease [11].

In spite of the constant interest of researchers in bat trypanosomes, questions about the means of their transmission between hosts and vectors are still the subject of debate [3]. A wide range of arthropods feed on the blood of bats: dipterans, true bugs, fleas, ticks and gamasine mites. Therefore, all of them theoretically can transmit trypanosomes [8,12,13]. For *T. dionisii*, bat bugs (some species of the heteropteran genus *Cimex*) are considered the main vectors [11,14,15]. In addition, it has been proposed that bat flies (family Nycteribiidae) could participate in the transmission of *T. dionisii* in Australia [16]. The analysis of the bat flies *Nycteribia schmidlii* in Europe (Italy, Hungary and Spain) revealed only some undescribed *Trypanosoma* spp. not directly related to *T. dionisii* [17].

In this work, we describe the first record of *T. dionisii* in Russia using molecular and morphological methods. We characterize the developmental stages of this flagellate in the bat-inhabiting gamasine mite *Steatonyssus periblepharus* and propose the latter to be a novel vector of *T. dionisii.*

## 2. Materials and Methods

### 2.1. Material Collection

**Figure 1:**
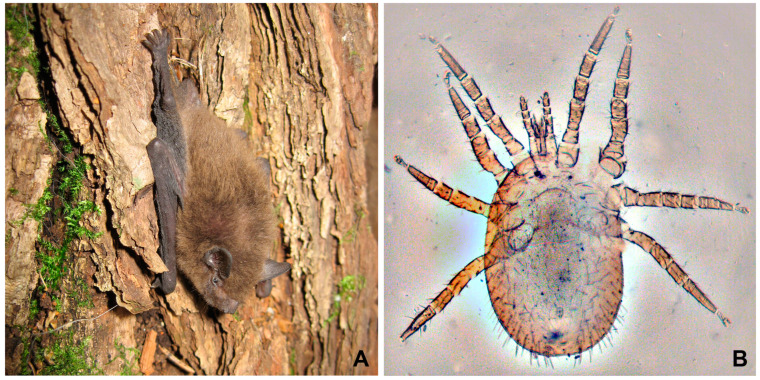
Putative host and vector of *T. dionisii* (**A**)—bat *Pipistrellus nathusii*; (**B**)—gamasine mite *Steatonyssus periblepharus* (preparation from the collection of the Zoological Institute of RAS).

Eleven bats were captured using 2 × 4 m mist nets in Sergievka Park (Petrodvortsovy District, St. Petersburg) in July 2022, including 10 individuals of *Pipistrellus nathusii* Keyserling and Blasius, 1839 (Nathusius’ pipistrelle) (Figure 1A) and 1 individual of *Myotis daubentonii* (Kuhl, 1817) (Daubenton’s bat). To collect *P. nathusii*, nets were installed near the colony located under the roof of a building. According to visual observations, there were about a hundred individuals of this species in the colony. *Myotis daubentonii* was caught on the territory of the park. All of the animals were examined at the place of capture. Mites and fleas were carefully collected with tweezers and placed in plastic 50 mL tubes individually for each host. After the collection of ectoparasites, all bats were immediately released into their natural habitat. A total of 18 fleas and 27 mites were collected.

The bats were investigated using non-invasive methods and were not removed from their habitat; therefore, no special permission was required for this study.

### 2.2. Analysis of Ectoparasites

The collected ectoparasites were anaesthetized with chloroform vapor and photographed. Fleas were dissected using syringe needles in a drop of 0.9% saline, which allowed analyzing separately their hemolymph and intestine. Meanwhile, mites, due to their extremely small size (1 mm or less), were simply crushed between a glass slide and a coverslip; therefore, it was not possible to identify the original localization of observed parasites. The contents of mites and fleas were examined under a Leica DM 2500 microscope (Leica Microsystems GmbH, Wetzlar, Germany). The remnants of the investigated arthropods were placed in 96% ethanol for subsequent analyses.

Mites were identified by methods of traditional taxonomy by arachnologist Dr. M. K. Stanyukovich, based on photos and the fragments of their crushed bodies. Identification of fleas was performed using molecular methods (see below).

### 2.3. Light Microscopy, Morphometry and Statistical Analysis

If trypanosomatids were detected in the investigated contents of an arthropod, smears were prepared from the sample, air-dried, fixed in 96% ethanol and stained with Romanowsky–Giemsa stain (30 min, pH 6.8). All microphotographs were taken using the abovementioned microscope equipped with a UCMOS14000KPA 14-Mpx camera (ToupTek, Hangzhou, China) using the 100× objective. The obtained photos were used for cell measurements performed with ImageJ v. 1.53 software [18].

The following characters were used for the morphological analysis of the identified *T. dionisii* forms and their subsequent comparison: cell length (L), width (W), size of the longitudinal axis of the nucleus (N), distance from the nucleus to the front end of the cell (NA), distance from the kinetoplast to the front end of the cell (KA) and length of the free part of the flagellum (F). The latter trait was relevant only for trypomastigotes, because in epimastigotes both organelles are located close to each other in most cases. Principal component analysis (PCA) using PAST 4.08 software [19] was applied to estimate the reliability of the identified differences separately for epi- and trypomastigote cells. The raw data were used to create a correlation matrix, from which two eigenvectors were extracted. The latter provided two axes, onto which the raw data were projected to produce two-dimensional plots of the morphotypes and characters.

### 2.4. DNA Isolation, PCR and Sequencing

The remains of fleas and trypanosome-positive mites were used for DNA extraction with the GeneJET Genomic DNA Purification Kit (ThermoFisher Scientific, Waltham, MA, USA) following the manufacturer’s protocol. The obtained DNA of fleas served for their molecular identification. An approximately 640 bp long fragment of cytochrome oxidase subunit I (COI) gene was amplified and sequenced using the standard barcoding primers LCOI1490 and HCOI2198 [20]. The obtained sequences were used as queries for searching the default database of the Barcode of Life Data System (http://boldsystems.org/ (accessed on 25 September 2023)).

The 18S rRNA gene sequence of trypanosomes was amplified from mite samples using trypanosomatid-specific primer pairs. It was either a single-step amplification with the primers 1127F and 1958R [21] producing a ~850 bp long fragment, or a nested PCR with the primers S762 and S763 [22] in the first round followed by TRnSSU-F2 and TRnSSU-R2 in the second round [23], resulting in an almost 2000 bp long product. The short PCR fragments were sequenced using the amplification primers, while the product of nested PCR was sequenced with internal primers S757, A757, 883F and 907R, as described previously [24]. The obtained sequences were deposited in GenBank under the accession numbers OR597300, OR597301, OR597494 and OR597495.

### 2.5. Phylogenetic Analyses

The obtained trypanosome sequences were compared to each other and used for blast search of related nucleotide sequences in the GenBank nr database. Two datasets were prepared: with minimal sequence lengths of 800 bp and of 500 bp, containing 54 and 231 sequences, respectively, including the haplotype revealed in this work and three outgroup species. Alignment of sequences was performed in MAFFT v. 7.490 [25] using the E-INS-I algorithm. The smaller dataset was then subjected to trimming with gBlocks as described elsewhere [26]. Maximum likelihood trees were inferred in IQ-TREE v. 2.2.0 [27] with automatic model selection. Edge support was estimated using 1000 replicates of the standard or ultrafast bootstrap method for the small and large datasets, respectively. The phylogenetic reconstruction for the small dataset was also performed using MrBayes v. 3.2.7 [28] run under the GTR + I + G model for 1,000,000 generations with every 100th generation sampled. Other parameters were set by default.

## 3. Results

### 3.1. Bat Ectoparasites and Presence of Trypanosomes in Them

A total of eighteen flea specimens were found in nine out of ten examined *Pipistrellus nathusii* individuals (Table 1). All these insects belonged to the species *Ischnopsyllus variabilis* (Wagner, 1898), as judged by COI gene sequences demonstrating 99.68–100% identity to those in the BOLD Systems database. Trypanosomes were not detected in any of the fleas.

Twenty-eight mites were found in eight bats and proved to be representatives of the superfamily Dermanyssoidea (suborder Gamasina, order Mesostigmata). One individual (from *Myotis daubentonii*) was identified as *Spinturnix* sp. (family Spinturnicidae), while the remaining 27 mites belonged to *Steatonyssus periblepharus* (family Macronyssidae, Figure 1B). The latter species was collected from seven out of ten examined *P. nathusii* and one *M. daubentonii*. Of all the arthropods analyzed here, trypanosomes were detected only in nine specimens of *S. periblepharus* collected from four individuals of pipistrelles. The percentage of trypanosome-bearing mites of this species constituted 33.3% (Table 1).

### 3.2. Molecular Identification of Trypanosomes and Phylogenetic Analysis

The amplification of the 18S rRNA gene was successful only with two samples from trypanosome-positive mites, apparently due to a scarcity of trypanosome cells. The obtained sequences proved to be identical to each other, i.e., represented a single haplotype. The blast search in GenBank retrieved *Trypanosoma dionisii* isolate P3 as the best hit (99,95% identity) with the only difference consisting in a single C-T transition. Thus, the trypanosome species was identified unambiguously.

**Figure 2:**
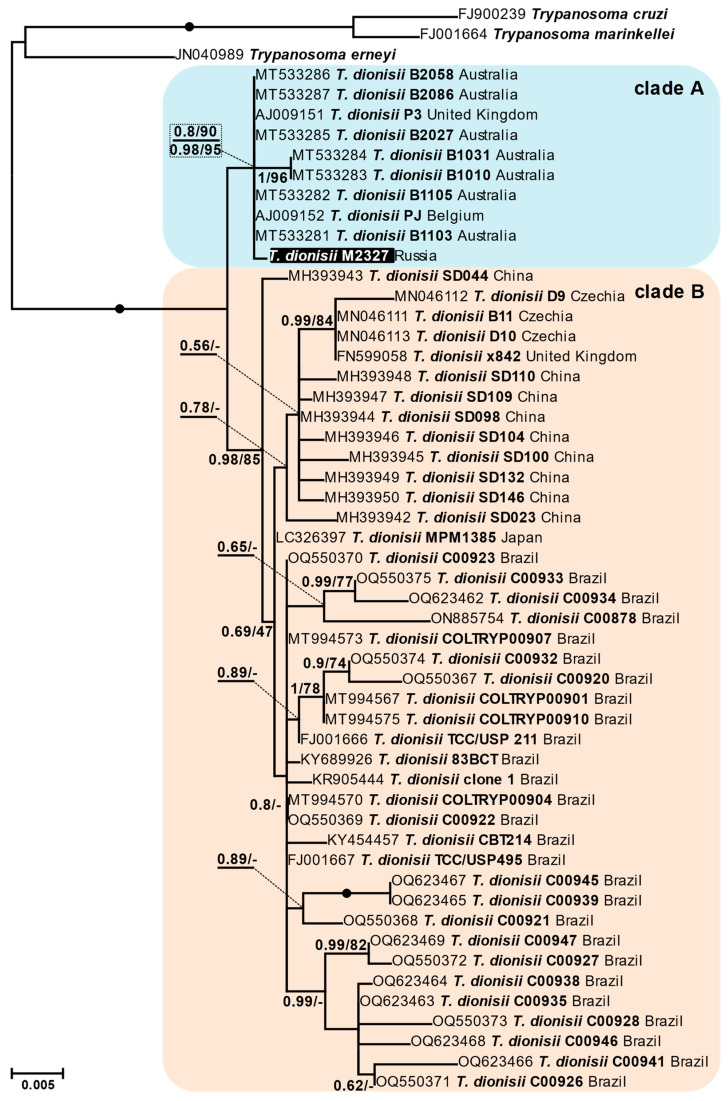
Maximum likelihood phylogenetic tree based on 18S rRNA gene sequences. The haplotype revealed in the studied isolates from mites is highlighted in black. Bayesian posterior probabilities and bootstrap values from maximum likelihood analysis are shown at branches (values below 0.5 or 50% are replaced with dashes or omitted). For the clade containing the haplotype from the mites, approximate Bayes and ultrafast bootstrap supports are additionally shown below the horizontal line (all four supports are boxed). The scale bar denotes the number of substitutions per site.

The phylogenetic analyses demonstrated that *T. dionisii* sequences were distributed between two groups of different sizes (Figure 2), which had previously been labelled as “clade A” and “clade B” [15,16]. While using the main dataset (at least 800 bp long sequences), the clade B had both high bootstrap support and posterior probability, the latter statistic was only moderate for the clade A, apparently due to the short length of some sequences. Additional testing of the corresponding branch using ultrafast bootstrap and approximate Bayes methods in IQ-TREE returned high supports for the clade A.

The 18S rRNA gene haplotype observed in mites was enclosed in the clade A along with the sequences of trypanosomes isolated in Australia, Belgium and the United Kingdom (Figure 2). According to the extended dataset, this clade also included *T. dionisii* isolates from Switzerland and South Africa (Figure S1). The other lineage, clade B, comprised sequences from Brazil, China, Japan, the United Kingdom and Czechia (Figure 2), as well as (according to the extended dataset) those from other countries in the Americas, Europe as well as Eastern and Southeastern Asia (Figure S1). Importantly, no members of the clade B have been found in Australia, while no American isolates were found in the clade A, despite their sequences in both datasets being predominant due to intensive and repeated sampling in previous studies.

The two clades of *T. dionisii* have significant genetic differences, suggesting that they represent distinct species. For example, the isolates PJ (AJ009152, clade A) and TCC/USP211 (FJ001666, clade B) have only 99.13% identity for the whole 18S rRNA gene as compared to 99.58% between *T. melophagium* (HQ664912) and *T. trinaperronei* (MN752212), two closely related, but undoubtedly distinct species of the subgenus *Megatrypanum* [29]. Other examples can be seen in the subgenus *Herpetosoma*: 99.72% identity between *T. rabinowitchae* (AY491765) and *T. blanchardi* (AY491764) or 99.26% between *T. microti* (AJ009158) and *T. otospermophili* (AB190228). Within the subgenus *Schizotrypanum* the identities between species are significantly lower (96–98.8%), which apparently indicates under-characterized diversity and rather conservative taxonomy. The latter is especially evident for *T. cruzi*, in which the identity between different strains (e.g., FJ900240 and AF359487) can be as low as 97.3%, which is consistent with the presence of several species-like lineages referred to as DTUs TcI-TcVI and Tcbat [30]. Known intraspecific variation in other species of the subgenus is much lower: with an identity of 99.68% in *T. marinkellei* (FJ001664 and AJ009150) and 99.82% in *T. erneyi* (JN040987 and JN040988), which does not contradict our assumption for *T. dionisii*.

### 3.3. Morphology

**Figure 3:**
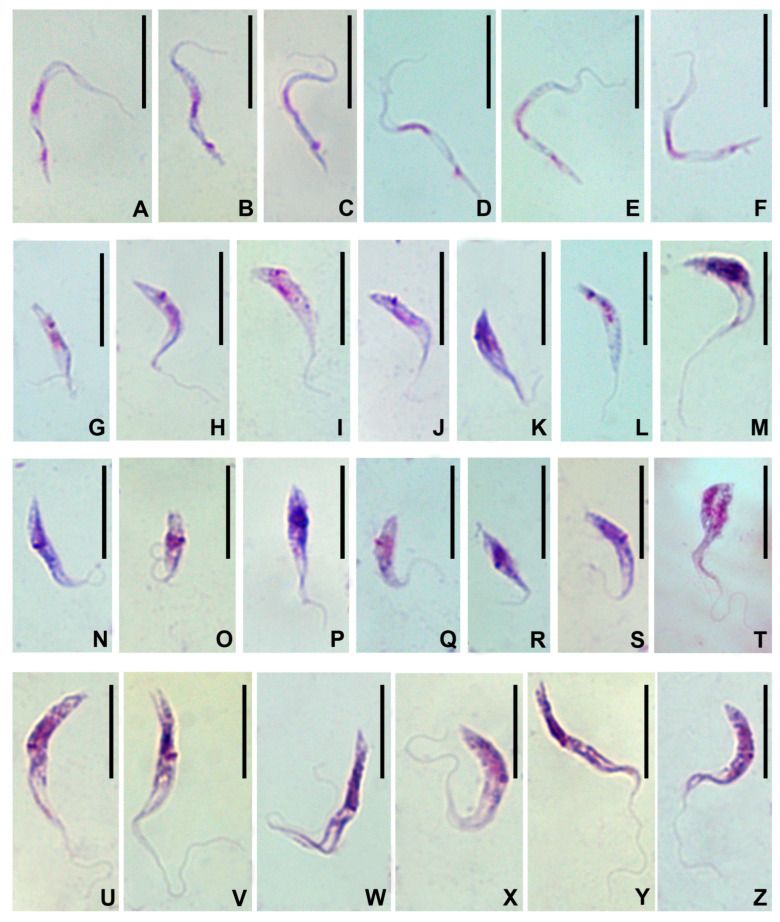
Morphs of *T. dionisii* from the mite *S. periblepharus*. (**A**–**F**)—slender metacyclic trypomastigotes; (**G**–**M**)—stumpy trypomastigotes; (**N**–**S**)—short epimastigotes; (**T**)—unequal division of epimastigotes; (**U**–**Z**)—long epimastigotes. The scale bar is 10 μm for all cases.

The cells of *Trypanosoma dionisii*, observed on the smears of the inner contents of mites, belonged to four distinct types. There were slender and stumpy trypomastigotes as well as short and long epimastigotes (Figure 3). The stumpy trypomastigotes were rarer than the other three types, which had comparable abundance.

The slender trypomastigotes (Figure 3A–F) appeared hook-shaped, had a tapered posterior end and measured on average 22 × 0.8 μm (Table 2). Their cytoplasm was very light, virtually transparent. The elongated nucleus was located in the middle of the cell and occupied its whole width. The kinetoplast was large, situated at a considerable distance from both the nucleus and the posterior end. The free part of the flagellum was very short or even completely indiscernible in some cells, while the undulating membrane was always quite conspicuous.

The stumpy trypomastigotes (Figure 3G–M) were almost half as short and twice as broad as the slender ones, measuring on average 12.6 × 1.7 µm (Table 2). They usually had a tapered posterior end and were sickle-shaped with a considerably darker cytoplasm as compared to the slender trypomastigotes. The nucleus was elongated and displaced posteriorly. The kinetoplast was localized near the posterior margin of the nucleus. The free part of the flagellum varied considerably in length, being undetectable in some cells and reaching almost the same length as the whole cell in others. The undulating membrane in most cells was hardly visible.

The short epimastigotes varied significantly in size and shape (Figure 3N–T) and measured on average 10.2 × 1.7 µm (Table 2). Their posterior endvaried from tapered to blunt and the cytoplasm was dense. In general, these cells looked similar to stumpy epimastigotes. However, their kinetoplast was located either laterally or, sometimes, anteriorly to the nucleus, but always very close to it. Both organelles were situated in the posterior half of the cell. In most cells the free part of the flagellum was short and the undulating membrane was hardly visible.

The long epimastigotes (Figure 3U–Z) were approximately twice as long as the short ones, while being of more or less the same width; on average they measured 19.6 × 1.7 μm (Table 2). Their posterior was conical or oblique, sometimes bifurcate. The nucleus was displaced posteriorly, while the kinetoplast was situated in a close proximity to the anterior margin thereof. The cytoplasm was generally dense and granular. In the anterior part of the cell, it stained unevenly: the area along the longitudinal axis was much lighter than the periphery and looked like a fission furrow. The free part of the flagellum sized about ¾ of the total cell length, while its attached portion formed an extended well-developed undulating membrane. The cytoplasm was dense and granular (Table 2, Figure 3U–Z).

The majority of cells on the smears were non-dividing. Division was observed only in epimastigotes (Figure 3T).

**Figure 4:**
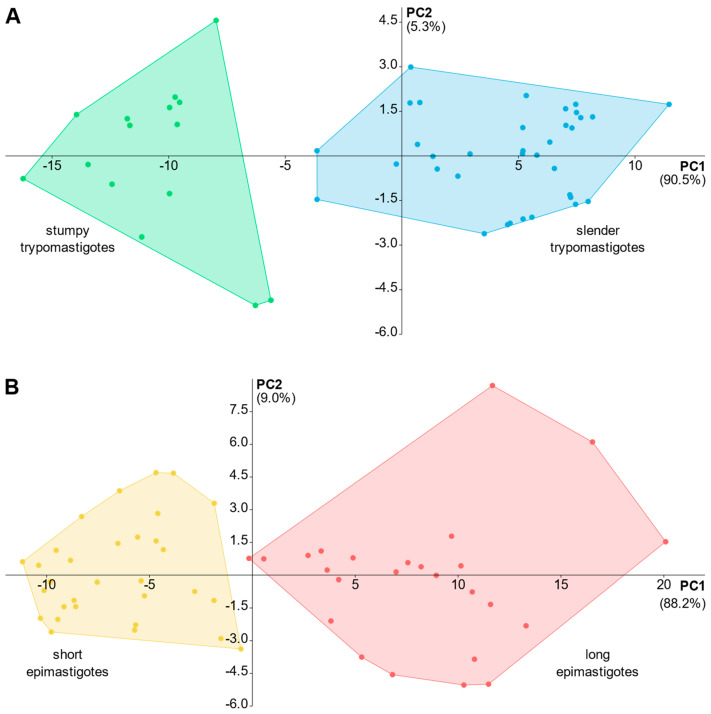
PCA scatterplots for *Trypanosoma dionisii* morphotypes. (**A**) Trypomastigotes. (**B**) epimastigotes. Values in parentheses correspond to the proportion of variance of the components.

The PCA analysis of *T. dionisii* cells was performed separately for epi- and trypomastigotes, since these basic morphotypes are unambiguously discriminated by the kinetoplast position. Our analysis revealed two factors with a maximum proportion of variance in the multivariate data (PC1 and PC2) responsible for over 95% of the variance (Table S1). The obtained scatterplots demonstrate that the observable differences between the cell types were reliable for their discrimination (Figure 4).

## 4. Discussion

The issue of vectors for *Trypanosoma dionisii*, as in the case of many other trypanosomes, is far from being well investigated. Although Cimicidae have been experimentally confirmed as vectors of this species in Europe [14], this was the only study on the topic. Indeed, some related trypanosomes have been also detected in the bugs of this family [4,12], indirectly supporting the view that bat bugs are important vectors of *T. dionisii*. However, the presence of *T. dionisii* in Australia [16], where the only available cimicids are the human-introduced synanthropic bed bugs *Cimex lectularius* and *C. hemipterus* [31], suggests that at least there it should be transmitted by different hematophagous arthropods. The example of *T. theileri complex* suggests that a single trypanosome species can have multiple vectors as evidenced by the presence of parasites with the same SSU rRNA gene haplotypes in tabanids, sandflies, mosquitoes and/or tsetse flies [32,33,34]. Such plasticity is generally not inherent to the trypanosomes, which are phylogenetically closer to *T. dionisii* (i.e., from the subgenera *Schizotrypanum* and *Aneza*). They are usually transmitted either by Triatominae or Cimicidae bugs, or both [3]. However, in older works one can find that *T. vespertilionis* was recorded not only in bat bugs, but also in mites, as well as suspected (based on the presence of epimastigotes in the gut) in bat-infesting nycteribiid flies [12]. However, considering that no molecular data are available for these records, the identity of the flagellates cannot be confirmed, so that these might not be *T. vespertilionis*.

The two clades of *T. dionisii*, as judged by the genetic distance between them, can be considered different species (see Results) and differ substantially in their geographic distribution. While the members of the clade A have been found in Europe, Africa and Australia, the representatives of the clade B originated from Europe, Asia and Americas. Although it can be argued that some areas were insufficiently sampled, this definitely cannot be a case for Brazil, wherefrom over 170 sequences were obtained in many independent studies [8,9,10,11,35,36,37,38,39,40,41,42,43]. Thus, the two clades/species have distinct geographic ranges with the only overlap documented so far being in Europe. Considering the high migration activity of bats, such a pattern suggests that these two clades/species have different vectors, which is in line with the abovementioned case of Australia. Regrettably, no sequence data are available for cimicid-transmitted *T. dionisii* in Europe, therefore we cannot assess whether it belongs to the same clade as the isolates documented here, or the other one.

The mites of the genus *Steatonyssus* (Acarina: Parasitiformes: Gamasina: Macronyssidae) are permanent specific parasites of chiropterans. Ecologically they occupy an intermediate position between nest-breeding and transient ectoparasites, which are characterized by a relatively short feeding time on the host and are more dependent on shelters than representatives of such genera as *Macronyssus* and *Spinturnix* [44]. In this respect, *Steatonyssus* spp. are close to cimicid bugs in the feeding mode and confinement to shelters. *Steatonyssus periblepharus* (Kolenati, 1858) is a trans-Palearctic species that parasitizes a wide range of bats of the families Vespertilionidae and Rhinolophidae, preferring mouse-eared bats (*Myotis* spp.) and pipistrelles (*Pipistrellus* spp.) [45].

Here we documented the first observation of *T. dionisii* in gamasine mites. The forms of trypanosomes that we revealed on the Giemsa-stained smears were dissimilar to those from the mammalian bloodstream, i.e., short and thin trypomastigotes with an almost terminally positioned kinetoplast [8,16]. However, they resembled the forms that had previously been documented in the intestine of *Cimex pipistrelli* experimentally infected with *T. dionisii* [14]. The only difference from the cited work consists in the fact that we discriminate two variants of epimastigotes instead of one. In general, polymorphism of developmental stages in vectors is typical for *T. cruzi*-like trypanosomes [12], although it is unclear what the functional differences between these multiple types could be. However, it is evident from our and previous observations that epimastigotes are proliferative, while two kinds of trypomastigotes are usually considered as metacyclic forms (i.e., infective for mammalian hosts) [14,46]. However, a recent study of metacyclogenesis in *T. cruzi* suggests that these can be successive developmental stages with the forms corresponding to our long epimastigotes in the beginning, followed by short epimastigotes, then stumpy epimastigotes and eventually becoming slender trypomastigotes, which are then the only genuine metacyclics [47].

In summary, the presence of characteristic developmental stages of *Trypanosoma dionisii* in *Steatonyssus periblepharus* strongly suggests that this mite represents a novel vector of this trypanosome, at least for its lineage known as clade A, which apparently represents a species distinct from its sister group, clade B. To ultimately confirm this, experiments with feeding *T. dionisii*-infected mites on bats are required.

## Figures and Tables

**Table 1:** Bat ectoparasites and trypanosome presence in them.

Bat Hosts			Ectoparasites (Trypanosome-Infected/Total)		
ID	Species	Sex and Age	*Steatonyssus periblepharus*	*Spinturnix* sp.	*Ischnopsyllus variabilis*
01	*Pipistrellus nathusii*	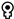 ad.	-	-	0/1
02	*Pipistrellus nathusii*	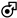 ad.	-	-	0/2
03	*Pipistrellus nathusii*	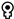 ad.	-	-	0/2
04	*Pipistrellus nathusii*	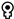 sad.	3/7	-	-
05	*Pipistrellus nathusii*	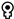 ad.	0/3	-	0/3
06	*Pipistrellus nathusii*	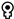 sad.	2/4	-	0/2
07	*Pipistrellus nathusii*	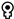 ad.	0/1	-	0/1
08	*Pipistrellus nathusii*	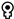 sad.	0/3	-	0/6
09	*Pipistrellus nathusii*	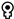 sad.	3/6	-	-
10	*Pipistrellus nathusii*	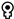 sad.	1/2	-	0/1
11	*Myotis daubentonii*	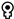 ad.	0/1	0/1	-
Total:			9/27	0/1	0/18

Abbreviations: ad.—adult; sad.—subadult. Dashes indicate no detected ectoparasites.

**Table 2:** Morphometry of Trypanosoma dionisii cells from Steatonyssus periblepharus.

Morphotype	N	Length	Width	Nucleus	N–A	K–A	Flagellum
Slender trypomastigotes	35	22.0 ± 0.4 (15.9–27.0)	0.8 ± 0.03 (0.5–1.2)	3.6 ± 0.1 (2.6–4.9)	12.0 ± 0.3 (5.5–15.1)	18.8 ± 0.4 (14.7–23.0)	2.3 ± 0.3 (0.0–5.9)
Stumpy trypomastigotes	15	12.6 ± 0.5 (9.2–16.3)	1.7 ± 0.1 (1.1–2.6)	1.8 ± 0.1 (1.1–2.3)	6.2 ± 0.3 (3.0–7.8)	9.5 ± 0.5 (6.1–12.0)	5.7 ± 0.7 (0.0–9.4)
Short epimastigotes	32	10.2 ± 0.4 (4.1–14.2)	1.7 ± 0.1 (1.0–2.5)	1.8 ± 0.1 (0.8–2.4)	5.3 ± 0.3 (1.8–9.1)	5.4 ± 0.3 (2.3–8.0)	6.2 ± 0.4 (3.1–12.3)
Long epimastigotes	26	19.6 ± 0.6 (14.7–26.2)	1.7 ± 0.1 (1.0–2.4)	1.9 ± 0.1 (1.2–2.5)	11.4 ± 0.5 (7.7–18.3)	10.8 ± 0.5 (6.9–16.1)	14.5 ± 0.7 (9.0–20.3)

N–A is the distance between the nucleus and the anterior end of the cell. K–A is the distance between the kinetoplast and the anterior end of the cell. All measurements are in μm (M ± SE (min–max)).

## Data Availability

Data used in this article can be found in the main text, supplementary material, or (in the case of sequences) in the GenBank database (see the list of accession numbers in Material and Methods).

## Supplementary Materials

The following supporting information can be downloaded at: https://www.mdpi.com/article/10.3390/microorganisms11122906/s1, Table S1: The loadings and proportion of variance for the two principal components. Figure S1: Maximum likelihood phylogenetic tree based on 18S rRNA gene sequences with a minimal length of 500 bp. The haplotype revealed here in mites and the clade of *T. dionisii* are highlighted in black and in light blue, respectively. Ultrafast bootstrap percentages are shown at branches for values > 50%. The scale bar denotes the number of substitutions per site.
