# Supplementary figures and images for: The Mite Steatonyssus periblepharus Is a Novel Potential Vector of the Bat Parasite Trypanosoma dionisii
Source: Microorganisms. 2023 Dec 1;11(12):2906. doi: 10.3390/microorganisms11122906 (PMC10745657; doi:10.3390/microorganisms11122906)

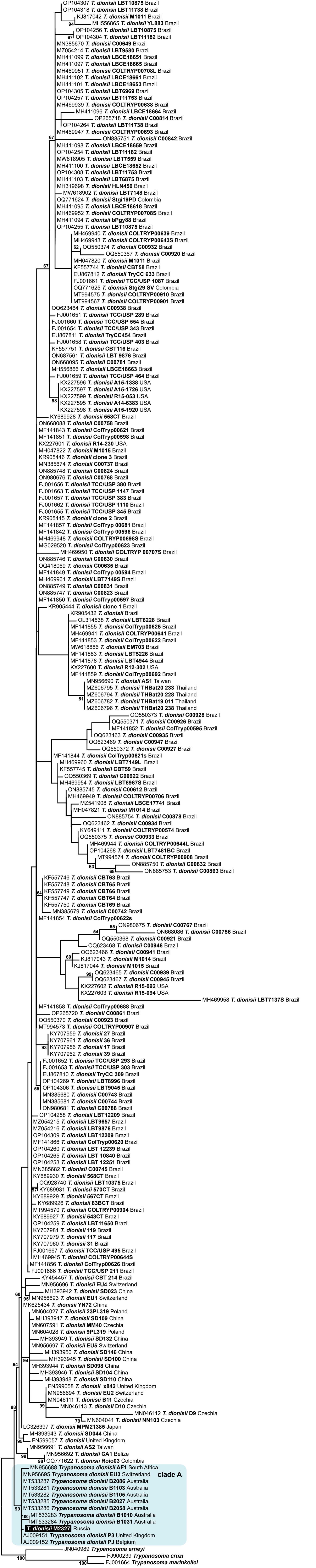

0.005

Supplement: Supplementary file 1 [file microorganisms-11-02906-s001.zip › Fig. S1.pdf]
